# Metronomic cyclophosphamide attenuates mTOR-mediated expansion of regulatory T cells, but does not impact clinical outcome in patients with metastatic renal cell cancer treated with everolimus

**DOI:** 10.1007/s00262-019-02313-z

**Published:** 2019-02-11

**Authors:** Inge M. Werter, Charlotte M. Huijts, Sinéad. M. Lougheed, Paul Hamberg, Marco B. Polee, Metin Tascilar, Maartje Los, John B. A. G. Haanen, Helgi H. Helgason, Henk M. Verheul, Tanja D. de Gruijl, Hans J. van der Vliet, for the Dutch WIN-O Consortium

**Affiliations:** 1 Department of Medical Oncology, Cancer Center Amsterdam, Amsterdam University Medical Centre, VU University Medical Centre, Vrije University, De Boelelaan 1117, 1081 HV Amsterdam, The Netherlands; 2 Department of Medical Oncology, Franciscus Gasthuis and Vlietland, Rotterdam, The Netherlands; 3 Department of Medical Oncology, Medical Center Leeuwarden, Leeuwarden, The Netherlands; 4 Department of Medical Oncology, Isala Clinics, Zwolle, The Netherlands; 5 Department of Medical Oncology, Saint Antonius Hospital, Nieuwegein, The Netherlands; 6 Department of Medical Oncology, Antoni van Leeuwenhoek Hospital, Amsterdam, The Netherlands; 7 Department of Medical Oncology, Haaglanden Medical Centre, The Hague, The Netherlands

**Keywords:** Everolimus, Cyclophosphamide, mRCC, Tregs, mTOR

## Abstract

**Introduction:**

Metastatic renal cell cancer (mRCC) patients have a median overall survival (mOS) of approximately 28 months. Until recently, mammalian target of rapamycin (mTOR) inhibition with everolimus was the standard second-line treatment regimen for mRCC patients, improving median progression-free survival (mPFS). Treatment with everolimus supports the expansion of immunosuppressive regulatory T cells (Tregs), which exert a negative effect on antitumor immune responses. In a phase 1 dose-escalation study, we have recently demonstrated that a low dose of 50 mg oral cyclophosphamide once daily can be safely combined with everolimus in mRCC patients and prevents the everolimus-induced increase in Tregs.

**Materials and methods:**

In a multicenter phase 2 study, performed in patients with mRCC not amenable to or progressive on a vascular endothelial growth factor (VEGF)-receptor tyrosine kinase inhibitor (TKI) containing treatment regimen, we assessed whether the addition of this metronomic dosing schedule of cyclophosphamide to therapy with everolimus could result in an improvement of progression-free survival (PFS) after 4 months of treatment.

**Results:**

Though results from this study confirmed that combination treatment effectively lowered circulating levels of Tregs, addition of cyclophosphamide did not improve the PFS rate at 4 months. For this reason, the study was abrogated at the predefined interim analysis.

**Conclusion:**

Although the comprehensive immunomonitoring analysis performed in this study provides relevant information for the design of future immunotherapeutic approaches, the addition of metronomic cyclophosphamide to mRCC patients receiving everolimus cannot be recommended.

## Introduction

Renal cell cancer (RCC) has been diagnosed in more than 84.000 new patients in the European Union each year and has resulted in almost 34.000 cancer deaths in 2012 [1]. Death due to RCC is mostly a consequence of metastatic disease, which occurs in 30% of patients at presentation and in an additional 30% of patients after initial nephrectomy [2]. Metastatic RCC (mRCC) is known to be resistant to chemotherapy. However, the prognosis of mRCC has greatly improved in the last decade with the registration of various novel therapeutics, resulting in a current median overall survival (mOS) of 28–29 months [3–6]. New drugs have been mostly tested in patients with clear cell mRCC, while papillary, chromophobic and oncocytic RCC and RCC of the collecting duct have been studied less due to their lower prevalence [7]. Until recently, first-line treatment of clear cell mRCC patients predominantly consisted of drugs that block the intracellular domain of the vascular endothelial growth factor (VEGF) receptor, such as sunitinib or pazopanib, resulting in a median progression-free survival (mPFS) of 11 months [8–10], or the combination of interferon-alfa (IFN-α) and bevacizumab, the latter binding circulating VEGF, which resulted in an mPFS of 8.5*–*10 months [11–14]. Since 2007, drugs targeting the mammalian target of rapamycin (mTOR) pathway have been registered for the treatment of mRCC. Temsirolimus represents a first-line treatment option in poor-risk mRCC patients, while everolimus became a standard Food and Drug Administration (FDA)-approved second-line treatment in 2009 [10, 15–18]. The mTOR pathway influences cell growth, proliferation and angiogenesis, and mTOR inhibitor everolimus leads to an mPFS of approximately 4 months when used as second-line treatment [16, 19]. Recently, the programmed cell death protein-1 (PD-1) checkpoint-inhibitor nivolumab, the c-Met and VEGF tyrosine kinase inhibitor (TKI) cabozantinib and the combination of lenvatinib (a multi kinase inhibitor) and everolimus were shown to be more effective compared to everolimus monotherapy and have thereby replaced everolimus as the standard second-line therapeutic approach in mRCC patients [3, 4, 20, 21]. In addition, combination therapy with PD-1 and cytotoxic T-lymphocyte-associated protein 4 (CTLA-4) immune checkpoint inhibitors nivolumab and ipilimumab was approved as a first-line treatment option for intermediate- and poor-risk patients [22].

One aspect potentially limiting the antitumor effect of mTOR inhibition by everolimus is their known stimulatory effect on regulatory T cells (Tregs) [23, 24]. Tregs are characterized by the expression of CD4, CD25 and the transcription factor forkhead box P3 (Foxp3), and are known to exert immunosuppressive effects, which can be beneficial in preventing overt autoimmunity, but can hamper the development of antitumor immune responses. Tumor cells or tumor-associated macrophages can produce ligands that selectively attract Tregs through C–C chemokine receptor (CCR) type 4, facilitating tumor cells to escape antitumor immunity [25, 26]. Studies have shown that the frequency of circulating as well as (peri)tumoral Tregs is negatively associated with survival in cancer patients, including mRCC patients [27–29].

We and others have shown that treatment with everolimus resulted in an expansion of peripheral blood Tregs [30, 31]. As we hypothesized that the undesirable everolimus-induced expansion of Tregs in mRCC patients could be counteracted, we co-administered cyclophosphamide, which is an alkylating agent of the nitrogen mustard type that is known to selectively deplete Tregs (and not helper or cytotoxic T cells) [32–34]. The effect of cyclophosphamide on Tregs is not completely understood; however, several mechanisms have been proposed, including (a) induction of a DNA repair defect, (b) reduction of the ATP and glutathion content of Tregs and (c) causing a lack in the expression of the ATP-binding cassette (ABC) transporters B1 (ABCB1) [33–36].

We first performed a phase 1 dose-escalation trial, in which we established the optimal dose of metronomic cyclophosphamide that, when combined with the standard once daily oral dose of 10 mg of everolimus, was safe, well tolerated and effectively reduced circulating levels of Tregs [37, 38]. In the present phase 2 study, we investigated whether the addition of the selected dose of metronomic cyclophosphamide would result in an improvement in mPFS as compared to everolimus monotherapy. In addition, immunomonitoring was performed to evaluate whether immune effects could be related to clinical outcome. The immunomonitoring performed in this study gives insight into the effects of mTOR inhibition and low-dose oral cyclophosphamide in cancer patients and thereby provides relevant information for the design of future treatments that incorporate or are based on mTOR inhibitors.

## Materials and methods

### Patients and treatment

The multicenter study was performed in medical centers that were part of the WIN-O (The Working group Immunotherapy of the Netherlands for Oncology) and included 29 patients of 18 years or older with clear cell mRCC who were not amenable to, or had progressed on, a VEGF receptor TKI regimen. As originally planned in the design of the study, 10 of the 25 patients had participated in the phase 1 part of this study, where they had been treated with the same treatment regimen as in the here reported phase 2 study. For a more extensive description of the inclusion and exclusion criteria of the study, we refer to the published study protocol [39]. Follow-up was until death or until the time of analysis (9 months after inclusion of the last patient). A pre-planned interim analysis was performed after 24 patients were treated for at least 4 months, to assess whether the primary objective, an increase of progression-free survival (PFS) at 4 months from 50 to 70% could be achieved. Since 12 out of 24 patients had progressed within the first 4 months of treatment, the study was terminated prematurely due to lack of efficacy. Secondary objectives that were studied included response rate, time to progression, overall survival and an assessment of the immunological effects of combination treatment.

Patients were treated with 10 mg everolimus and 50 mg cyclophosphamide orally, both once daily continuously. In case of severe toxicity, dose reductions were allowed according to protocol. Adverse events (AE) were defined in accordance with the International Conference on Harmonisation (ICH) Guideline for Good Clinical Practice (ICH E6:1.2). Severity of clinical AE was graded according to the National Cancer Institute Common Toxicity Criteria (CTC) grading system version 3.0 (NCI-CTCAE). Dose-limiting toxicities (DLT) were toxicities attributable to combination therapy within the first 28 days of therapy and defined as febrile neutropenia, neutropenic infection, other grade ≥ 3 hematological toxicity, pneumonitis, nausea, vomiting, diarrhea, fatigue or any other grade ≥ 3 AE that, despite appropriate supportive care, failed to recover to grade ≤ 1 within 7 days [39]. Patients were evaluated at baseline and then every 4 weeks until the end of study treatment by means of history, physical examination and laboratory evaluation (hematology and chemistry). Moreover computed tomography scans (CT scan) of chest and abdomen were made at baseline and thereafter every 8 weeks. The objective response rate (ORR) was assessed clinically and radiologically, using Response Evaluation Criteria In Solid Tumors (RECIST, version 1.1).

### Immunomonitoring

Immunomonitoring was performed at baseline and 4 weeks after the start of the study treatment period. Peripheral blood mononuclear cells (PBMC) were isolated by Lymphoprep (Axis-Shield, Oslo, Norway) density-gradient centrifugation, cryopreserved in liquid nitrogen, thawed and subsequently stained for 30 min at 4 °C with labeled antibodies in phosphate-buffered saline (PBS) supplemented with 0.1% bovine serum albumin (BSA) and 0.02% sodium azide. Based on the immunomonitoring results of the previously performed phase 1 study [37, 38], the following immune cell subsets were selected for monitoring in the present phase 2 study: regulatory T cells (Tregs, CD4^+^CD25^hi^FoxP3^hi^), cytotoxic T cells (CTL, CD3^+^CD8^+^), B lymphocytes (CD19^+^), myeloid dendritic cells (cDC1, BDCA3^+^CD14^−^CD11c^+^ and cDC2, BDCA1^+^CD19^−^CD14^−^CD11c^+^) and plasmacytoid dendritic cells (pDC, BDCA2^+^CD11c^−^CD123^+^), immunoregulatory (CD56^bright^CD16^dim^) and cytotoxic (CD56^dim^CD16^+^) natural killer cells (NK), and granulocytic (Lin^−^CD14^−^CD33^+^HLA^−^DR^−^) and monocytic (Lin^−^CD14^+^HLA^−^DR^−^) myeloid derived suppressor cells (MDSC).

The following antibodies were used: FITC-labeled antibodies against IgG1, CD4, CD14, CD16, BDCA1, BDCA2 and BDCA3; PE-labeled antibodies against IgG1, CD8, CD19, CD40, CD56, CD86 and CD123; PerCP-labeled antibodies against IgG1, CD3 and CD4; APC-labeled antibodies against IgG1, CD3, CD11c, CD25 and PD-1 (all these antibodies were obtained from BD Biosciences, New Jersey, USA). Intracellular stainings were performed after fixation and permeabilization using a fixation/permeabilization kit according to the manufacturer’s protocol (eBioscience, Massachusetts, USA). The labeled antibodies used for intracellular stainings were PE-labeled IgG1, IgG2a, CTLA-4 and Ki-67 (all BD Biosciences, New Jersey, USA). FoxP3 was stained with anti-FoxP3 mAbs, either PCH101 PE (eBioscience, Massachusetts, USA) or 259D Alexa Fluor 488 (Biolegend, San Diego, USA). All cells were analyzed on a BD FACS Calibur and analyzed using Kaluza Analysis Software (Beckman Coulter).

### Statistical analysis

Paired *t* tests were used to determine the statistical significance of differences between time points or groups. PFS was defined as the time from baseline until progression or death. Overall survival (OS) was defined as the time from baseline until death. Both PFS and OS were analyzed using Kaplan–Meier curves. Correlations were measured using Pearson correlation coefficient. Findings were considered statistically significant when *p* values were ≤ 0.05, as indicated with asterisks (*p ≤ 0.05, **p < 0.01, ***p < 0.001). Statistical analyses were performed using GraphPad Prism 6.0 software.

## Results

### Baseline patient characteristics

The study included 29 patients with clear cell mRCC who were treated at 12 different centers in the Netherlands between November 2013 and October 2016. Of these 29 patients, 25 patients were followed according to protocol; 4 of 29 patients were excluded within the first 2 weeks of the start of treatment. Three of them withdrew consent and one patient had inadvertently taken an inappropriate dose of cyclophosphamide. Patient characteristics are shown in Table 1. Of the 25 patients included for study analysis, 60% were male, the median age of the study group was 66 years and 80% could be categorized in the favorable or intermediate IMDC risk group (prognostic model according to the International Metastatic Renal Cell Carcinoma Database Consortium, IMDC). The mean amount of white blood cells (WBC) was 6.4 × 10^9^/L (± 0.38 SEM) and mean amount of lymphocytes was 1.45 × 10^9^/L (± 0.13 SEM).

**Table 1:** Baseline characteristics

Characteristic		Study group (*N* = 25)
Sex—no. (%)		
Male		15 (60)
Female		10 (40)
Median age—year (range)		66 (48 − 78)
ECOG performance status—no. (%)		
0		11 (44)
1		11 (44)
2		1 (14)
Unknown		2 (8)
IMDC risk group—no. (%)		
Favorable		5 (20)
Intermediate		15(60)
Poor		4 (16)
Unknown		1 (4)
Median time from initial diagnosis to metastastic disease—mo. (range)		12.5 (0 − 174.5)
Median time from metastastic disease to start study treatment—mo. (range)		20 (1 − 54.5)
Site of metastases—no. (%)		
Lung		18 (72)
Lymph nodes		19 (76)
Bone		6 (24)
Kidney		4 (16)
Other*		8 (32)
Number of metastatic sites—no. (%)		
1		5 (20)
2		9 (36)
3		5 (20)
≥4		5 (20)
Unknown		1 (4)
Previous systemic cancer therapy—no. (%)		
Sunitinib		13 (52)
Pazopanib		8 (32)
Interferon + bevazucimab		1 (4)
Sorafenib		1 (4)
Previous antiangiogenic regimens—no. (%)		
0 or unknown		6 (24)
1		15 (60)
>1		4 (16)

*Adrenal, liver, soft tissue, subcutaneous, peritoneum, breast

### Treatment efficacy and safety

The median time of treatment of patients was 4.2 months (range 0.5*–*11 months). Two patients (8%) still received treatment at the time of study termination, and all other patients had discontinued study medication due to progression (*n* = 19, 76%) or unacceptable toxicity (*n* = 4, 16%). Median follow-up was 7.9 months (range 0.5–21 months), based on time until death (*n* = 13, 52%) or until time of analysis (*n* = 12, 48%).

**Fig. 1:**
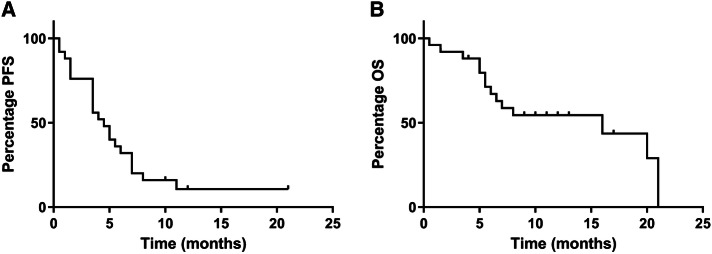
Percentage Progression-free survival and overall survival on treatment. **a** Median PFS is 4.5 months (range 0.5–21 months). At 4 months, 48% (*n* = 12) of 25 patients had progressive disease. **b** Median OS is 16 months (range 0.5–20 months). OS data are preliminary, as 11 patients (44%) were still alive at the end of the follow-up period (range 4–20 months). Data were analyzed using a Kaplan–Meier curve

At the predefined interim analysis, it became evident that the primary objective of the study, an increase of PFS at 4 months from 50 to 70%, could not be reached. At 4 months, 48% (*n* = 12) of 25 patients had progressive disease. mPFS and mOS were 4.5 months (range 0.5*–*21 months) and 16 months (range 0.5*–*20 months), respectively (Fig. 1a, b). Three patients did not show signs of progression at the time of analysis (range 10*–*21 months) and 11 patients were still alive at the end of the follow-up period (range 10*–*21 months). The best clinical outcome was stable disease (SD) in 72% (*n* = 18) of the cases. Progressive disease (PD) was observed in 28% (*n* = 7) of the patients. No partial or complete responses were observed.

Overall, combination treatment was reasonably well tolerated. A total of 168 different AEs was reported, an average of 6.7 per patient. No grade 4 or 5 toxicities were observed. The most common (> 30%) treatment-related toxicities were fatigue (*n* = 11; 44%), anemia (*n* = 10; 40%), pneumonitis (*n* = 10; 40%), anorexia (*n* = 8; 32%) and hypercholesterolemia (*n* = 8; 32%) (Table 2). A total of 18 treatment-related grade 3 AEs were reported in 13 (52%) patients. Grade 3 toxicities included fatigue, anemia, pneumonitis and leukocytopenia. Three patients (12%) endured a DLT related to study medication within 28 days after the start of treatment, i.e., hematuria grade 3, nausea grade 3 and mucositis grade 3. In the case of the patient with hematuria, this resolved upon discontinuation of cyclophosphamide and the patient continued with everolimus treatment until disease progression. For the patient with nausea, study medication was interrupted and, due to rapid disease progression, not reintroduced. In the patient with mucositis, this resolved after a 14 day interruption of study medication and did not recur upon reintroduction of study combination therapy.

**Table 2:** Treatment-related toxicities, reported in > 10% of patients

Event	Any grade *N* (%)	Grade 1 *N* (%)	Grade 2 *N* (%)	Grade 3 *N* (%)
*Constitutional*				
Fatigue	11 (44)	3 (12)	5 (20)	2 (8)
Anorexia	8 (32)	2 (8)	6 (24)	
Malaise	6 (24)	2 (8)	2 (8)	2 (8)
Pain	4 (16)	2 (8)	2 (8)	
Fever/chills/flu	3 (12)	3 (12)		
Sweating/flushes	3 (12)	3 (12)		
*Dermatology*				
Rash	6 (24)	4 (16)	2 (8)	
Pruritus	3 (12)	2 (8)	1 (4)	
*Gastrointestinal*				
Nausea	7 (28)	4 (16)	2 (8)	1 (4)
Mucositis	7 (28)	4 (16)	2 (8)	1 (4)
Stomatitis	6 (24)	4 (16)	1 (4)	1 (4)
Diarrhea	5 (20)	5 (20)		
Constipation	3 (12)	1 (4)	2 (8)	
Dysgeusia	3 (12)	2 (8)	1 (4)	
*Laboratory*				
Anemia	10 (40)	1 (4)	7 (28)	2 (8)
Hypercholesteremia	8 (32)	2 (8)	6 (24)	
Hyperglycaemia	6 (24)	1 (4)	4 (16)	1 (4)
Leukocytopenia	6 (24)	1 (4)	3 (12)	2 (8)
Hypertriglyceridemia	5 (20)	2 (8)	2 (8)	1 (4)
Thrombocytopenia	5 (20)	4 (16)		1 (4)
Electrolyte disturbance*	4 (16)	4 (16)		
*Respiratory*				
Pneumonitis	10 (40)	3 (12)	5 (20)	2 (8)
Dyspnea	7 (28)	5 (20)	2 (8)	
Cough	6 (24)	5 (20)	1 (4)	

*Hyponatremia, hypokalemia, hypercalcemia

### Immune monitoring

Based on the immunomonitoring results of the previously performed phase 1 study, a selective panel of immune cell subsets and ratios between immune cell subsets were analyzed in this phase 2 study: total CD3^+^ T cells, CD3^+^CD4^+^ conventional T-helper cells (Tconv), CD3^+^CD8^+^ CTL, Tregs, effector–suppressor T cell ratio or CD8:Treg ratio, defined as the ratio between CD8^+^ effector T cells and suppressive Tregs), immunoregulatory and cytotoxic NK cells, cDC1, cDC2 and pDC [31, 37, 38]. In the present study, the total amount of PBMC was 2.01 × 10^9/^/L ± 0.13 (mean ± standard error of mean (SEM) at baseline and 1.86 × 10^9/^/L ± 0.14 after 4 weeks of treatment (not significant, NS). The total lymphocyte count was 1.45 × 10^9/^/L ± 0.13 at baseline and 1.25 × 10^9/^/L ± 0.11 after 4 weeks of treatment (NS).

#### T cell subsets

**Fig. 2:**
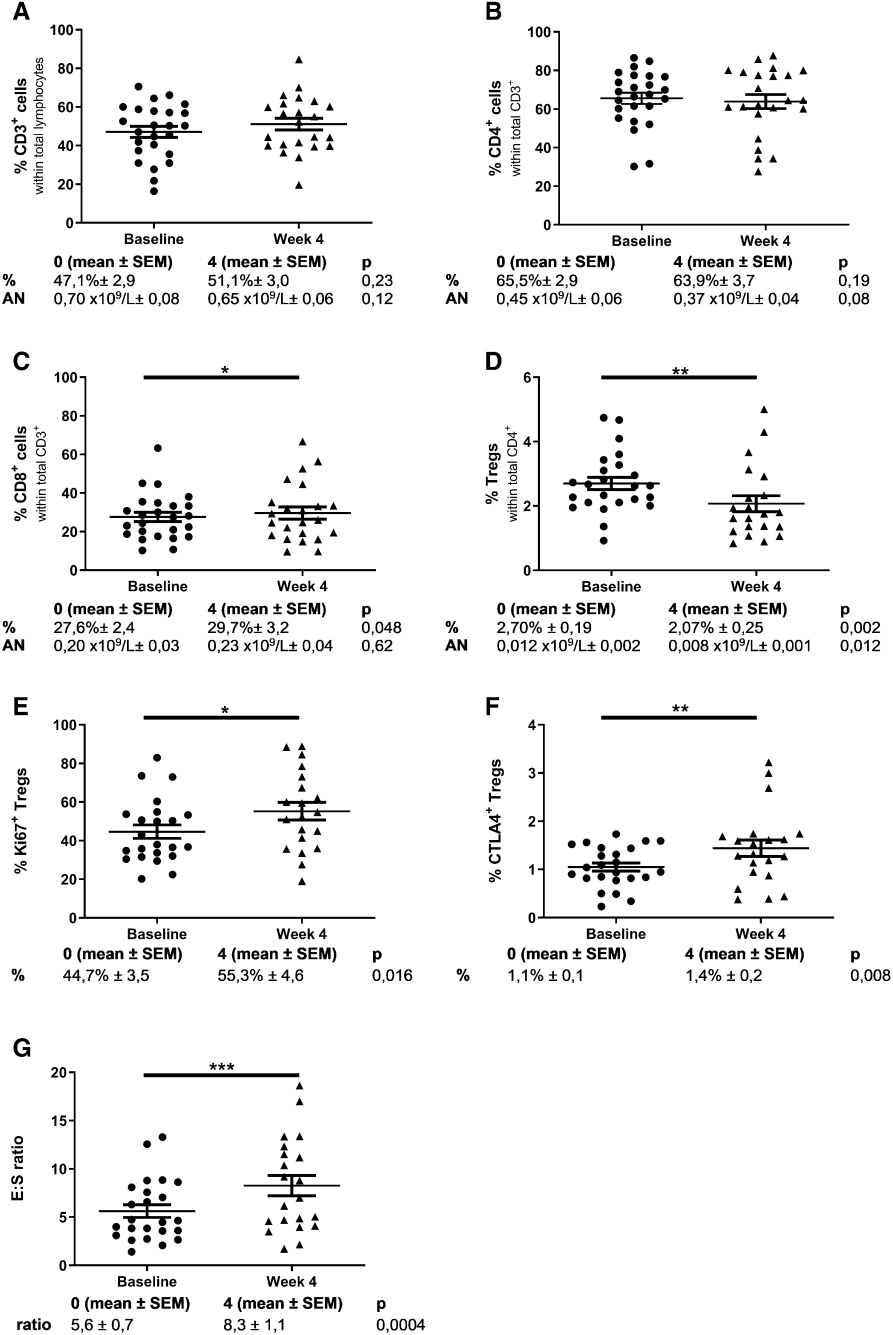
Change in lymphocyte subsets between baseline and 4 weeks of treatment. **a** Percentage of T cells (CD3^+^) in total lymphocytes. **b** Percentage of T helper cells (CD4^+^) in total CD3^+^ cells. **c** Percentage of cytotoxic T cells (CD8^+^) in total CD3^+^ cells. **d** Percentage of regulatory T cells (CD4^+^CD25^hi^FoxP3^hi^) in total CD4^+^ cells. **e** Percentage of Ki-67^+^ (Ki-67^+^CD4^+^CD25^hi^FoxP3^hi^) in regulatory T cells. **f** Percentage of CTLA4^+^ (CTLA4^+^CD4^+^CD25^hi^FoxP3^hi^) in regulatory T cells. **g** E:S ratio. Effector (CD8^+^):suppressor (CD4^+^CD25^hi^FoxP3^hi^) ratio. Data were analyzed using paired *t* tests. *p ≤ 0.05, **p < 0.01, ***p < 0.001

Neither the frequency nor absolute numbers (AN) of CD3^+^ T cells in the total lymphocyte population changed significantly during the first 4 weeks of treatment (Fig. 2a). Also, the frequency as well as the absolute numbers of circulating CD4^+^ T cells did not change significantly (Fig. 2b). Of interest, a small but statistically significant increase in CD8^+^ CTL was observed in frequency, and a similar, but not significant, trend was seen in absolute numbers (Fig. 2c).

The frequency and absolute numbers of circulating regulatory T cells (CD4^+^CD25^hi^FoxP3^hi^) was found to significantly decrease from baseline to week 4 (Fig. 2d), confirming our previous observations. Of note, although the frequency of circulating Tregs decreased during the first 4 weeks of treatment, expression of the proliferation marker Ki-67 and the inhibitory CTLA-4 receptor in Tregs significantly increased (Fig. 2e, f).

As the ratio between CD8^+^ effector T cells and suppressive Tregs (E:S ratio) can have a prognostic impact [40], changes in this ratio were also assessed. As illustrated in Fig. 2g, the E:S ratio significantly increased from baseline to week 4, reflective of a change in the relative distribution between T cell subsets toward a more favorable balance when considering antitumor immune responses.

#### Changes in natural killer (NK) cell populations

**Fig. 3:**
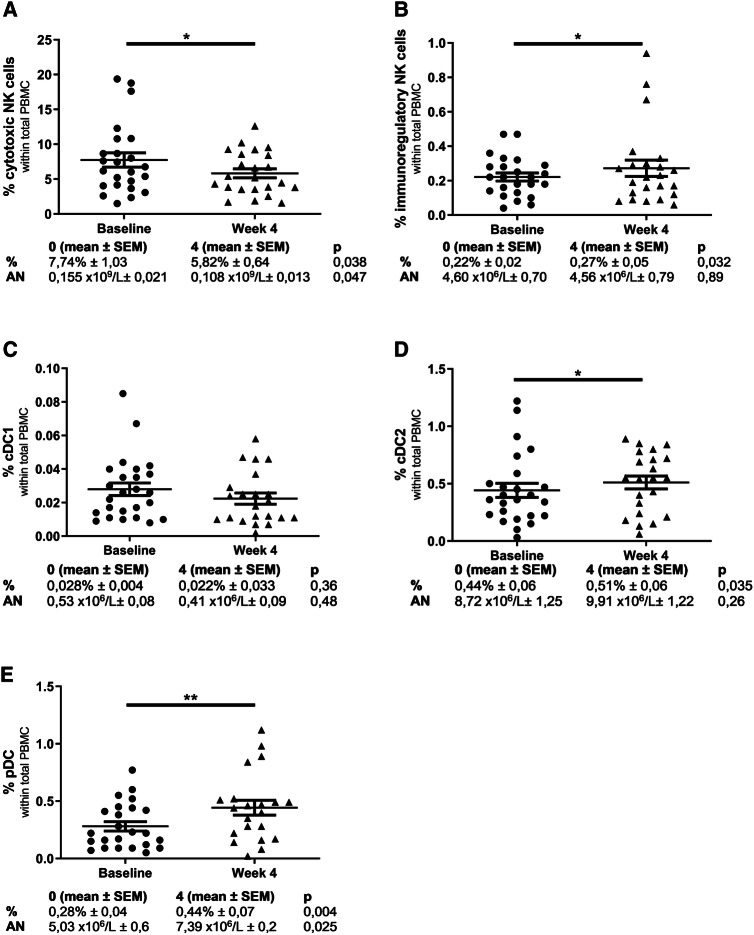
Change in NK and DC cell populations between baseline and 4 weeks of treatment. **a** Percentage of cytotoxic NK cells (CD56^dim^CD16^+^) in PBMC. **b** Percentage of immunoregulatory NK cells (CD56^bright^CD16^−^) in PBMC. **C**. Percentage of cDC1 (BDCA3^+^CD14^−^CD11c^+^) in PBMC. **d** Percentage of cDC2 (BDCA1^+^CD19^−^CD14^−^ CD11c^+^) in PBMC. **e** Percentage of pDC (CD11c^−^BDCA2^+^CD123^+^) in PBMC. Data were analyzed using paired *t* tests. *p ≤ 0.05, **p < 0.01, ***p < 0.001

After 4 weeks of treatment, a shift within the NK cell population occurred. There was a significant decline in both the frequency and absolute number of cytotoxic (CD56^dim^CD16^+^) NK cells (Fig. 3a). In contrast, the immunoregulatory (CD56^bright^CD16^−^) NK cell population significantly increased in frequency, though not in absolute numbers (Fig. 3b). Overall, the effect of combination treatment of cyclophosphamide and everolimus on the NK cell balance was opposite to the effect observed with T cells and resulted in a more immunoregulatory NK cell profile.

#### Circulating dendritic cell subsets

Several blood dendritic cell subsets were monitored, including myeloid dendritic cells (cDC1 and cDC2) and plasmacytoid dendritic cells (pDC). After 4 weeks of treatment, a small, but non-significant, decrease in cDC1 cells was observed both in frequency and in absolute numbers (Fig. 3c). A significant increase in the frequency, but not in absolute numbers of cDC2 was observed (Fig. 3d). For pDC an increase was demonstrated in frequency as well as in absolute numbers (Fig. 3e). In addition to the frequency of circulating DC subsets, their expression of DC activation markers was monitored (data not shown). The activation status of cDC1, cDC2 and pDC did not significantly change, as measured by the expression of CD40, CD86 and CD123 (the latter only for pDC, data not shown).

#### Immunomonitoring and correlation with clinical outcome

**Fig. 4:**
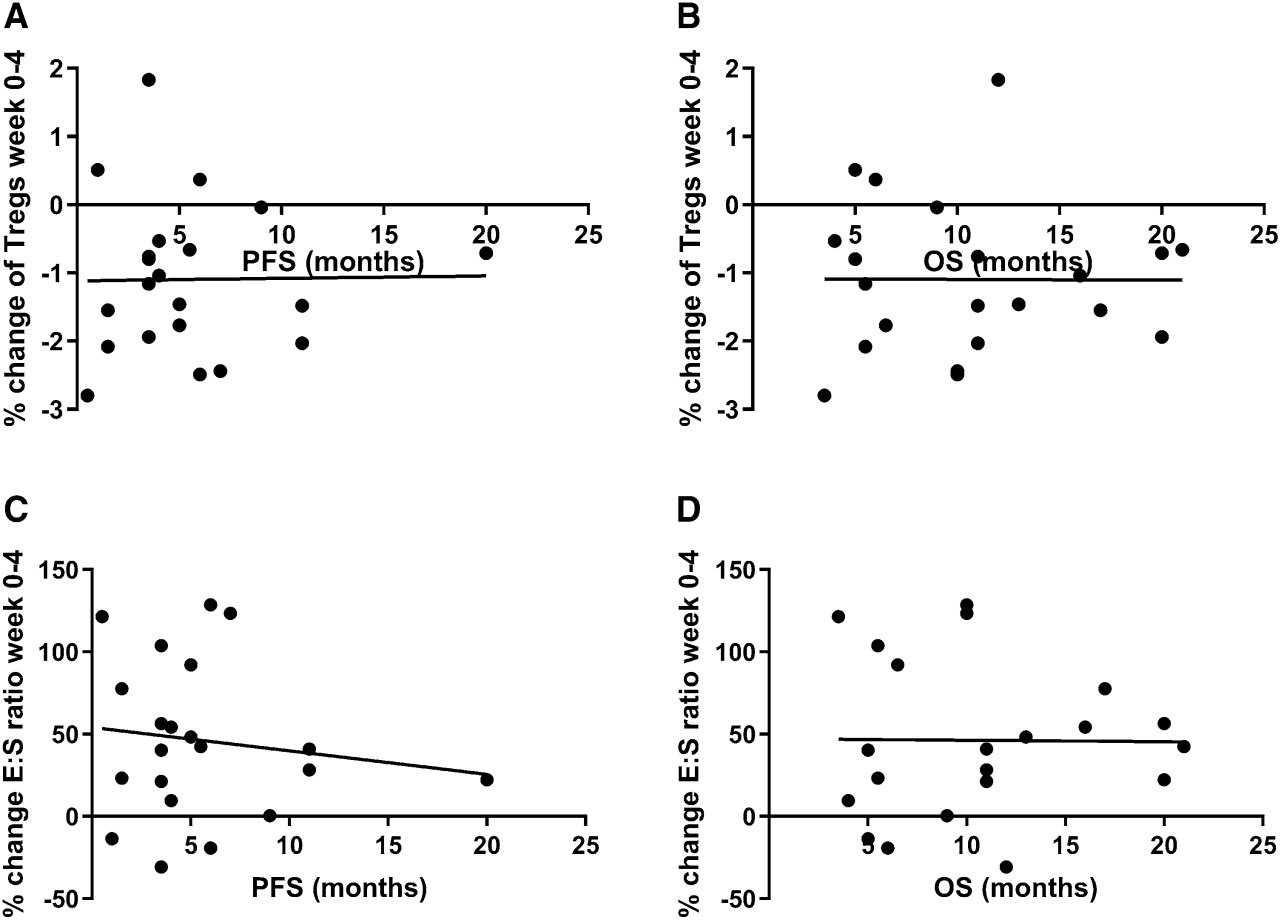
Correlation between survival and changes in Treg frequency and E:S ratio between baseline and 4 weeks of treatment. **a**. Correlation between PFS (months) and relative percentual change in the percentage of Tregs between baseline and 4 weeks of treatment, Pearson *r* = 0.014 (*p* = 0.95). **b** Correlation between OS (months) and relative percentual change in the percentage of Tregs between baseline and 4 weeks of treatment, Pearson *r* = − 0.004 (*p* = 0.99). **c** Correlation between PFS (months) and relative percentual change of E:S ratio between baseline and 4 weeks of treatment, Pearson *r* = − 0.133 (*p* = 0.564). **d** Correlation between OS (months) and relative percentual change of E:S ratio between baseline and 4 weeks of treatment, Pearson *r* = − 0.011 (*p* = 0.963). Data were analyzed using Pearson correlation coefficient. Relative percentual change is the percentage of week 4 minus the percentage at baseline, divided by the percentage at baseline

Overall, combination therapy with low-dose oral cyclophosphamide and everolimus did not improve the clinical outcome of patients when compared to everolimus monotherapy. However, as the combination of cyclophosphamide and everolimus resulted in a significant decrease in Tregs and an increase in the E:S ratio, we explored whether changes in these parameters could be related to the outcome. For this purpose, possible correlations between survival (both PFS and OS) and the percentage of Tregs at baseline, the E:S ratio at baseline, the percentage of Tregs at week 4, the E:S ratio at week 4, the percentual change of Tregs from baseline to week 4 and the percentual change in E:S ratio between baseline and week 4 were assessed (Fig. 4a–d, not all correlations shown). Altogether, a correlation between PFS or OS and either the frequency of Tregs, the E:S ratio or changes herein could not be demonstrated. However, it is noteworthy that in the three patients with the longest PFS (i.e., >1 year), both a decrease in the percentage of Tregs and an increase in the E:S ratio between baseline and week 4 was observed.

## Discussion

Overall, the results from the present phase 2 study demonstrate that, while the addition of low-dose oral cyclophosphamide to everolimus treatment in patients with clear cell mRCC effectively prevents the everolimus-induced increase in immunosuppressive Tregs, this does not result in clinical benefit. As the predefined goal of the study of improving the PFS rate at 4 months from 50 to 70% was not reached, the study was terminated at the preplanned interim analysis.

Several studies have aimed to lower the amount of Tregs in cancer patients by the administration of cyclophosphamide, with varying results [32, 33, 41, 42]. As there is controversy on the optimal dose and schedule of cyclophosphamide when aiming for Treg depletion and no such data are available for the combination of cyclophosphamide and everolimus, we first performed a phase 1 study in which we set out to determine the optimal Treg-depleting dose of cyclophosphamide when combined with the standard dose of everolimus [37, 38]. In our phase 1 study, continuous once daily oral dosing of 50 mg of cyclophosphamide proved to be most effective in lowering the percentage of Tregs, and therefore this dose was selected for the present phase 2 study. Of note, while we confirmed that once daily oral administration of 50 mg of cyclophosphamide in this phase 2 trial resulted in a reduction in circulating Treg levels after 4 weeks of treatment, an increase in expression of the proliferation marker Ki-67 was observed in Tregs and this was accompanied by an upregulation of the expression of the inhibitory CTLA-4^+^ molecule on Tregs. In accordance with these increased Ki-67 levels, a small rebound in Treg levels was observed after 8 weeks of treatment in the phase 1 part of our study [37, 38]; in the phase 2 part of the study these measurements were not done after 8 weeks. Our observations are in line with results of a study by Ge et al., demonstrating a similar rebound in circulating Treg levels after an initial decrease during the first 14 days of treatment with 50 mg cyclophosphamide once daily in breast cancer patients. This was accompanied by an increase in the proliferative activity of Tregs with a maintained suppressive capacity [33]. Of note, whereas Ge et al. reported a correlation between the temporary reduction in Treg levels and improved clinical outcome, our study showed no relation between a reduction in Tregs and the outcome. Clearly, the clinical impact of cyclophosphamide-induced effects on Tregs may not only differ per selected cyclophosphamide treatment schedule, but also per tumor type as well as any concomitant treatment such as everolimus in our study. Various mechanisms have been implicated as causative factors for the Treg depletion that is observed, such as the mechanisms mentioned earlier, but also low expression of aldehyde dehydrogenase 1 (ALDH1), inhibition of indoleamine 2,3-dioxygenase (IDO), ATP depletion, CCR2 expression and effects on MDSC; however, it is unknown why the effect of cyclophosphamide on Tregs appears to be temporary [43–45].

In our study, the combination of everolimus and cyclophosphamide did not affect the frequency of circulating CD4^+^ T cells and actually resulted in an increased frequency of CD8^+^ T cells with a concomitant increase in the E:S ratio. Though the E:S ratio has previously been reported to be significantly associated with improved survival in cancer patients, we did not find a correlation between E:S ratio and survival [40]. Of note, the association between E:S ratio and survival was mostly reported in studies performing analyses in (peri)tumoral tissues instead of peripheral blood [40, 46, 47]. In our study, no serial tumor biopsies were performed precluding similar analyses.

Overall, the balance between the monitored immune cell subsets in our study appeared to shift toward a more robust antitumor immune profile, as illustrated by the selective reduction in the percentage of Tregs and increase in effector CD8^+^ CTLs as well as blood DC subsets. This did, however, not translate into an enhanced clinical efficacy of combination treatment with everolimus and cyclophosphamide, which may reside in induced changes in the NK cell population, as an increase in the CD56^bright^ immunoregulatory NK cell population and a decrease in the CD56^dim^ cytotoxic NK cell population were observed. The change in the balance between both NK cell populations can be attributed to cyclophosphamide, as an opposite effect (i.e., a decrease in immunoregulatory NK cells and an increase in cytotoxic NK cells) was observed in the phase 1 patients treated with everolimus monotherapy [31]. A possible explanation might be the preferential apoptosis of CD56^dim^ cytotoxic NK cells, as postulated by Bauernhofer et al. [48].

It will be interesting to explore whether therapeutic approaches that can counteract this putative detrimental effect of cyclophosphamide on the NK cell population can improve clinical antitumor activity of the combination of everolimus and cyclophosphamide. For example, the TKI axitinib and the anti-epileptic drug valproic acid have been reported to in vitro increase expression of NKG2D ligands on tumor cells, thereby increasing their susceptibility to NK cell and γδ T cell recognition [49, 50]. Alternatively, very low doses of recombinant IL-2 and IFN-α could be considered, as these were reported to increase NK cell numbers in vivo, albeit that these consisted mainly of the CD56^bright^ cell subset and they will probable increase Treg numbers as well [51]. Potential drawbacks for such triple combination treatment regimens are related to an increased risk of toxicity. For example, studies combining a VEGF TKI with an mTOR inhibitor in mRCC were mostly terminated prematurely as a result of significant toxicity [52, 53].

As stated before, among others, the PD-1 checkpoint inhibitor nivolumab has replaced everolimus as the standard second-line therapy in mRCC patients. Future studies investigating whether nivolumab can efficiently counteract the immunosuppressive effects observed with everolimus monotherapy may be considered and could potentially result in more potent antitumor activity than either treatment alone.

In conclusion, results from the present phase 2 clinical study demonstrate that addition of low-dose metronomic cyclophosphamide to everolimus can effectively prevent the everolimus-induced increase in Tregs in mRCC patients and in addition results in an increased frequency of CD8^+^ CTL, cDC2 and pDC. The Treg-depleting effect diminished over time (as demonstrated in the phase 1 study [37, 38]), which may be related to the observed increase in Ki-67^+^ levels in Tregs and was accompanied by a minor increase in Treg CTLA4^+^ expression, a decline of cytotoxic NK cells and an increase of immunoregulatory NK cells. Overall, the immunomodulatory effects of the combination of metronomic cyclophosphamide and everolimus did not translate into an altered clinical outcome as measured by the percentage of patients progression free after 4 months of therapy. The comprehensive immunomonitoring analysis performed in this study provides relevant insight for the rational design of future therapeutic approaches in mRCC and other malignancies such as neuroendocrine tumors, in which mTOR inhibitors are also used as anti-cancer therapeutics.

## Abbreviations

ABCB1: ATP-binding cassette (ABC) transporters B1
ALDH1: Aldehyde dehydrogenase 1
AN: Absolute numbers
APC: Allophycocyanin
CCMO: Central Committee on Research Involving Human Subjects
CCR: C-C chemokine receptor
CT scan: Computed tomography scan
CTC: Common Toxicity Criteria
DLT: Dose-limiting toxicities
E:S ratio: Effector to suppressor ratio
FDA: Food and Drug Administration
ICH: International Conference on Harmonization
IMDC: International Metastatic RCC Database Consortium
METC: Medical Ethical Committee
mOS: Median overall survival
mPFS: Median progression-free survival
mRCC: Metastatic renal cell cancer
mTOR: Mammalian target of rapamycin
NCI-CTCAE: National Cancer Institute Common Toxicity Criteria grading system
ORR: Objective response rate
PD: Progressive disease
RCC: Renal cell carcinoma
SD: Stable disease
Tconv: Conventional T-helper cells
TKI: Tyrosine kinase inhibitor
WIN-O: Dutch Working Group on Immunotherapy of Oncology
